# (5*R*,8*R*)-2-(3,8-Dimethyl-2-oxo-1,2,4,5,6,7,8,8a-octa­hydro­azulen-5-yl)acrylic acid (rupestonic acid)

**DOI:** 10.1107/S1600536808001402

**Published:** 2008-01-23

**Authors:** Haji Akber Aisa, Jian-Ping Yong, Qiao-Ying Lv, Tao Wu

**Affiliations:** a Xinjiang Technical Institute of Physics and Chemistry, Chinese Academy of Science, Urumqi 830011, People’s Republic of China; b Graduate School of Chinese Academy of Science, Beijing 100039, People’s Republic of China

## Abstract

The title compound, C_15_H_20_O_3_, crystallizes with two independent mol­ecules in the asymmetric unit. In both mol­ecules, the seven-membered ring adopts a chair conformation. In the crystal structure, inter­molecular O—H⋯O hydrogen bonds link the mol­ecules into chains extending in the [201] direction. The absolute configuration was assigned on the basis of the starting materials.

## Related literature

For related crystal structures, see: Oberti *et al.* (1983 ▶). For biological activities of sesquiterpenes, see: Endo *et al.* (1979 ▶); Iguchi *et al.* (1986 ▶); Kubo *et al.* (1992 ▶); Delgado *et al.* (1991 ▶)
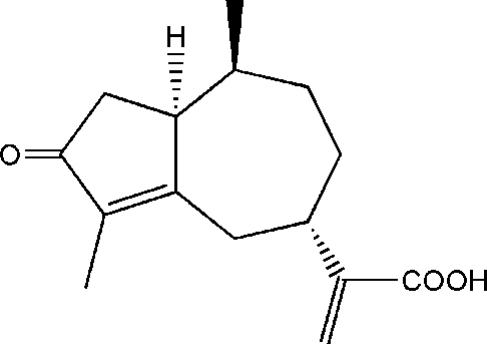


## Experimental

### 

#### Crystal data

C_15_H_20_O_3_
*M* *_r_* = 248.31
Monoclinic, 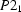

*a* = 9.5295 (19) Å
*b* = 9.4821 (19) Å
*c* = 15.047 (3) Å
β = 98.36 (3)°
*V* = 1345.2 (5) Å^3^
*Z* = 4
Mo *K*α radiation
μ = 0.08 mm^−1^
*T* = 293 (2) K
0.29 × 0.08 × 0.08 mm

#### Data collection

Rigaku R-AXIS RAPID IP area-detector diffractometer
Absorption correction: multi-scan (*ABSCOR*; Higashi, 1995 ▶) *T*_min_ = 0.976, *T*_max_ = 0.993
13040 measured reflections
3254 independent reflections
2012 reflections with *I* > 2σ(*I*)
*R*_int_ = 0.067

#### Refinement

*R*[*F* ^2^ > 2σ(*F* ^2^)] = 0.053
*wR*(*F* ^2^) = 0.111
*S* = 1.02
3254 reflections
343 parameters
1 restraint
H atoms treated by a mixture of independent and constrained refinement
Δρ_max_ = 0.14 e Å^−3^
Δρ_min_ = −0.16 e Å^−3^

### 

Data collection: *RAPID-AUTO* (Rigaku, 2004 ▶); cell refinement: *RAPID-AUTO*; data reduction: *RAPID-AUTO* program(s) used to solve structure: *SHELXTL* (Sheldrick, 2008 ▶); program(s) used to refine structure: *SHELXTL*; molecular graphics: *SHELXTL*; software used to prepare material for publication: *SHELXTL*.

## Supplementary Material

Crystal structure: contains datablocks I, global. DOI: 10.1107/S1600536808001402/cv2378sup1.cif

Structure factors: contains datablocks I. DOI: 10.1107/S1600536808001402/cv2378Isup2.hkl

Additional supplementary materials:  crystallographic information; 3D view; checkCIF report

## Figures and Tables

**Table 1:** Hydrogen-bond geometry (Å, °)

*D*—H⋯*A*	*D*—H	H⋯*A*	*D*⋯*A*	*D*—H⋯*A*
O2—H2*A*⋯O6	0.91 (6)	1.80 (6)	2.699 (4)	166 (5)
O5—H5*A*⋯O3^i^	0.90 (5)	1.76 (5)	2.658 (4)	171 (5)

Symmetry code: (i) 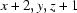.

**Fig. 1.**
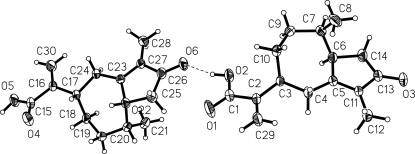
The content of asymmetric unit of (I) showing the atomic numbering and displacement ellipsoids drawn at the 30% probability level. Hydrogen bonds are shown as dashed lines.

## supplementary crystallographic information

### Comment

Rupestonic acid (the title compound) is a sesquiterpene with multifunctional groups, isolated from the Artemisia Rupestris *L*. (Chinese name is Yizhihao). Sesquiterpenes always exhibit considerable biological activities such as antiinflammatory (Endo *et al.*, 1979), ichtyotoxic and cytotoxic (Iguchi *et al.*, 1986), molluscicidal activities (Kubo *et al.*, 1992; Delgado *et al.*, 1991). Our researching groups have tested the title compound against the herpes simplex type 1, herpes simplex type 2 (HSV-1, HSV-2) and the influenza A3,B virus. The results showed that it exhibits higher activity against influenza B virus (TC50=258.69ug/ml, IC50=28.74ug/ml). We report here the crystal structure of the title compound (I).

In (I) (Fig. 1), all bond lengths and angles are normal and in a good agreement with those reported previously (Oberti *et al.*, 1983). The title compound crystallizes with two independent molecules in the asymmetric unit. In both molecules, the seven-membered ring adopts a chair conformation. In the crystal, the intermolecular O—H···O hydrogen bonds (Table 1) link the molecules into chains extended in direction [201].

### Experimental

The dry Artemisia Rupestris *L*. (Chinese name is Yizhihao) was extracted with ethanol, the extraction was evaporated under reduced pressure. The rusidue was purified by the silicon gel column chromatography (EtOAc/ petroleum ether: 5:1–2:1) to obtain the title compound (I). Crystals suitable for X-ray diffraction analysis were obtained by re-crystallization of (I) in acetone/petral ether (1:1 V/V) repetitiously at room temperature.

### Refinement

All H atoms were found on difference maps. Atoms H2A, H5A, H9A and H9A were isotropically refined. The remaining H atoms were placed in calculated positions, with C—H = 0.93–0.98 Å, and included in the final cycles of refinement using a riding model, with *U*_iso_(H) = 1.2 (1.5 for methyl) times *U*_eq_(C). In the absence of any significant anomalous scatterers in the compound, the 1052 Friedel pairs were merged before the final refinement.

### Crystal data

**Table:**

C_15_H_20_O_3_	*F*(000) = 536
*M**_r_* = 248.31	*D*_x_ = 1.226 Mg m^−^^3^
Monoclinic, *P*2_1_	Mo *K*α radiation, λ = 0.71073 Å
Hall symbol: P 2yb	Cell parameters from 6207 reflections
*a* = 9.5295 (19) Å	θ = 6.0–55.0°
*b* = 9.4821 (19) Å	µ = 0.08 mm^−^^1^
*c* = 15.047 (3) Å	*T* = 293 K
β = 98.36 (3)°	Needle, colorless
*V* = 1345.2 (5) Å^3^	0.29 × 0.08 × 0.08 mm
*Z* = 4	

### Data collection

**Table:**

Rigaku R-AXIS RAPID IP area-detector diffractometer	3254 independent reflections
Radiation source: Rotating Anode	2012 reflections with *I* > 2σ(*I*)
graphite	*R*_int_ = 0.067
ω oscillation scans	θ_max_ = 27.5°, θ_min_ = 3.1°
Absorption correction: multi-scan (*ABSCOR*; Higashi, 1995)	*h* = −12→12
*T*_min_ = 0.976, *T*_max_ = 0.993	*k* = −11→12
13040 measured reflections	*l* = −19→19

### Refinement

**Table:**

Refinement on *F*^2^	Primary atom site location: structure-invariant direct methods
Least-squares matrix: full	Secondary atom site location: difference Fourier map
*R*[*F*^2^ > 2σ(*F*^2^)] = 0.053	Hydrogen site location: inferred from neighbouring sites
*wR*(*F*^2^) = 0.111	H atoms treated by a mixture of independent and constrained refinement
*S* = 1.02	*w* = 1/[σ^2^(*F*_o_^2^) + (0.0543*P*)^2^] where *P* = (*F*_o_^2^ + 2*F*_c_^2^)/3
3254 reflections	(Δ/σ)_max_ < 0.001
343 parameters	Δρ_max_ = 0.14 e Å^−^^3^
1 restraint	Δρ_min_ = −0.16 e Å^−^^3^

### Special details

**Table:**

Geometry. All e.s.d.'s (except the e.s.d. in the dihedral angle between two l.s. planes) are estimated using the full covariance matrix. The cell e.s.d.'s are taken into account individually in the estimation of e.s.d.'s in distances, angles and torsion angles; correlations between e.s.d.'s in cell parameters are only used when they are defined by crystal symmetry. An approximate (isotropic) treatment of cell e.s.d.'s is used for estimating e.s.d.'s involving l.s. planes.
Refinement. Refinement of *F*^2^ against ALL reflections. The weighted *R*-factor *wR* and goodness of fit *S* are based on *F*^2^, conventional *R*-factors *R* are based on *F*, with *F* set to zero for negative *F*^2^. The threshold expression of *F*^2^ > σ(*F*^2^) is used only for calculating *R*-factors(gt) *etc*. and is not relevant to the choice of reflections for refinement. *R*-factors based on *F*^2^ are statistically about twice as large as those based on *F*, and *R*- factors based on ALL data will be even larger.

### Fractional atomic coordinates and isotropic or equivalent isotropic displacement parameters (Å^2^)

**Table:**

	*x*	*y*	*z*	*U*_iso_*/*U*_eq_	
O1	0.1814 (3)	0.3739 (4)	0.2549 (2)	0.1099 (14)	
O2	0.1916 (3)	0.4857 (3)	0.12807 (18)	0.0632 (7)	
O3	−0.6320 (3)	0.5450 (4)	−0.18584 (17)	0.0883 (11)	
O4	1.1007 (3)	0.4719 (3)	0.65333 (19)	0.0753 (8)	
O5	1.2819 (3)	0.6146 (4)	0.6438 (2)	0.0726 (8)	
O6	0.4647 (2)	0.5556 (3)	0.18400 (17)	0.0685 (8)	
C1	0.1251 (4)	0.4058 (4)	0.1813 (3)	0.0539 (10)	
C2	−0.0184 (3)	0.3600 (4)	0.1407 (2)	0.0418 (8)	
C3	−0.0794 (3)	0.4117 (3)	0.0475 (2)	0.0395 (7)	
H3A	−0.0651	0.5140	0.0465	0.047*	
C4	−0.2392 (3)	0.3848 (4)	0.0280 (2)	0.0484 (9)	
H4A	−0.2538	0.2841	0.0200	0.058*	
H4B	−0.2804	0.4122	0.0807	0.058*	
C5	−0.3195 (3)	0.4575 (4)	−0.0511 (2)	0.0438 (8)	
C6	−0.2531 (3)	0.5444 (4)	−0.1181 (2)	0.0475 (8)	
H6A	−0.2052	0.6255	−0.0866	0.057*	
C7	−0.1458 (3)	0.4665 (4)	−0.1671 (2)	0.0507 (9)	
H7A	−0.1267	0.5285	−0.2161	0.061*	
C8	−0.2072 (5)	0.3311 (5)	−0.2112 (3)	0.0745 (12)	
H8A	−0.1375	0.2862	−0.2417	0.112*	
H8B	−0.2896	0.3529	−0.2536	0.112*	
H8C	−0.2331	0.2687	−0.1660	0.112*	
C9	−0.0031 (4)	0.4419 (5)	−0.1081 (3)	0.0541 (10)	
C10	−0.0028 (4)	0.3477 (4)	−0.0261 (2)	0.0482 (9)	
H10A	0.0946	0.3278	−0.0008	0.058*	
H10B	−0.0478	0.2588	−0.0452	0.058*	
C11	−0.4614 (3)	0.4519 (4)	−0.0694 (2)	0.0515 (9)	
C12	−0.5604 (4)	0.3782 (6)	−0.0172 (3)	0.0790 (15)	
H12A	−0.5440	0.4097	0.0440	0.118*	
H12B	−0.5446	0.2783	−0.0193	0.118*	
H12C	−0.6564	0.3989	−0.0428	0.118*	
C13	−0.5085 (4)	0.5330 (5)	−0.1502 (2)	0.0579 (10)	
C14	−0.3832 (4)	0.5989 (5)	−0.1823 (3)	0.0626 (10)	
H14A	−0.3889	0.7009	−0.1797	0.075*	
H14B	−0.3775	0.5710	−0.2437	0.075*	
C15	1.1608 (3)	0.5541 (4)	0.6110 (2)	0.0511 (9)	
C16	1.1042 (3)	0.5984 (4)	0.5178 (2)	0.0458 (8)	
C17	0.9465 (3)	0.5744 (4)	0.4913 (2)	0.0415 (8)	
H17A	0.9257	0.4770	0.5069	0.050*	
C18	0.8648 (3)	0.6722 (4)	0.5459 (2)	0.0510 (9)	
H18A	0.9221	0.6884	0.6038	0.061*	
H18B	0.8526	0.7623	0.5153	0.061*	
C19	0.7194 (3)	0.6196 (5)	0.5620 (2)	0.0545 (9)	
H19A	0.7314	0.5273	0.5899	0.065*	
H19B	0.6846	0.6824	0.6048	0.065*	
C20	0.6060 (3)	0.6081 (4)	0.4799 (2)	0.0484 (8)	
H20A	0.5217	0.5698	0.5016	0.058*	
C21	0.5632 (4)	0.7506 (5)	0.4404 (3)	0.0631 (11)	
H21A	0.5390	0.8116	0.4868	0.095*	
H21B	0.4827	0.7403	0.3945	0.095*	
H21C	0.6408	0.7907	0.4148	0.095*	
C22	0.6444 (3)	0.5018 (4)	0.4090 (2)	0.0458 (8)	
H22A	0.6818	0.4149	0.4387	0.055*	
C23	0.7508 (3)	0.5621 (3)	0.3543 (2)	0.0390 (7)	
C24	0.9026 (3)	0.5920 (4)	0.3903 (2)	0.0450 (8)	
H24A	0.9234	0.6881	0.3746	0.054*	
H24B	0.9615	0.5304	0.3599	0.054*	
C25	0.5173 (3)	0.4672 (5)	0.3372 (2)	0.0571 (9)	
H25A	0.5093	0.3662	0.3274	0.069*	
H25B	0.4298	0.5016	0.3551	0.069*	
C26	0.5476 (3)	0.5405 (4)	0.2543 (2)	0.0501 (9)	
C27	0.6940 (3)	0.5903 (4)	0.2692 (2)	0.0422 (8)	
C28	0.7624 (4)	0.6651 (4)	0.1996 (3)	0.0598 (10)	
H28A	0.8567	0.6293	0.1998	0.090*	
H28B	0.7668	0.7643	0.2126	0.090*	
H28C	0.7078	0.6500	0.1416	0.090*	
C29	−0.0845 (4)	0.2777 (5)	0.1914 (3)	0.0642 (11)	
H29A	−0.0407	0.2523	0.2484	0.077*	
H29B	−0.1752	0.2448	0.1702	0.077*	
C30	1.1901 (4)	0.6522 (5)	0.4661 (3)	0.0745 (14)	
H30B	1.2861	0.6625	0.4877	0.089*	
H30C	1.1551	0.6801	0.4078	0.089*	
H2A	0.281 (6)	0.505 (6)	0.156 (4)	0.13 (2)*	
H5A	1.310 (5)	0.600 (5)	0.703 (3)	0.095 (16)*	
H9A	0.038 (4)	0.530 (4)	−0.085 (2)	0.053 (10)*	
H9B	0.064 (3)	0.408 (3)	−0.146 (2)	0.045 (9)*	

### Atomic displacement parameters (Å^2^)

**Table:**

	*U*^11^	*U*^22^	*U*^33^	*U*^12^	*U*^13^	*U*^23^
O1	0.071 (2)	0.175 (4)	0.069 (2)	−0.038 (2)	−0.0345 (17)	0.046 (2)
O2	0.0440 (14)	0.0778 (19)	0.0615 (17)	−0.0130 (14)	−0.0139 (13)	0.0067 (15)
O3	0.0444 (15)	0.156 (3)	0.0570 (17)	0.0192 (17)	−0.0173 (12)	0.006 (2)
O4	0.0705 (17)	0.088 (2)	0.0608 (18)	−0.0119 (17)	−0.0140 (14)	0.0196 (17)
O5	0.0468 (15)	0.107 (2)	0.0570 (19)	−0.0091 (15)	−0.0175 (13)	0.0051 (17)
O6	0.0454 (14)	0.098 (2)	0.0551 (17)	−0.0105 (14)	−0.0164 (12)	−0.0039 (16)
C1	0.044 (2)	0.066 (2)	0.048 (2)	0.0027 (18)	−0.0050 (18)	0.002 (2)
C2	0.0367 (17)	0.045 (2)	0.0414 (19)	0.0039 (15)	−0.0034 (14)	0.0019 (16)
C3	0.0309 (16)	0.0446 (18)	0.0411 (19)	0.0009 (13)	−0.0010 (13)	−0.0020 (15)
C4	0.0371 (17)	0.062 (2)	0.043 (2)	−0.0046 (16)	−0.0052 (14)	0.0043 (17)
C5	0.0365 (17)	0.055 (2)	0.0383 (19)	−0.0006 (16)	−0.0010 (14)	−0.0051 (17)
C6	0.0417 (17)	0.051 (2)	0.047 (2)	0.0049 (15)	−0.0006 (15)	0.0064 (17)
C7	0.0506 (19)	0.062 (2)	0.0388 (19)	−0.0016 (18)	0.0026 (15)	0.0049 (18)
C8	0.089 (3)	0.085 (3)	0.046 (2)	0.004 (3)	−0.003 (2)	−0.014 (2)
C9	0.045 (2)	0.069 (3)	0.050 (2)	0.010 (2)	0.0107 (18)	0.004 (2)
C10	0.0398 (17)	0.053 (2)	0.050 (2)	0.0096 (16)	−0.0016 (16)	0.0045 (18)
C11	0.0329 (17)	0.082 (3)	0.0371 (19)	−0.0007 (17)	−0.0021 (14)	−0.0076 (19)
C12	0.040 (2)	0.141 (5)	0.053 (2)	−0.019 (2)	−0.0010 (18)	0.000 (3)
C13	0.0418 (19)	0.084 (3)	0.044 (2)	0.0127 (19)	−0.0054 (16)	−0.004 (2)
C14	0.050 (2)	0.076 (3)	0.057 (2)	0.012 (2)	−0.0069 (17)	0.016 (2)
C15	0.0392 (19)	0.057 (2)	0.052 (2)	0.0039 (17)	−0.0078 (16)	0.003 (2)
C16	0.0329 (15)	0.058 (2)	0.043 (2)	0.0003 (16)	−0.0042 (14)	−0.0023 (17)
C17	0.0320 (15)	0.055 (2)	0.0353 (17)	0.0000 (15)	−0.0033 (13)	−0.0019 (16)
C18	0.0346 (17)	0.067 (2)	0.048 (2)	0.0031 (17)	−0.0060 (15)	−0.0116 (18)
C19	0.0463 (19)	0.075 (3)	0.043 (2)	0.0037 (18)	0.0092 (16)	−0.0038 (18)
C20	0.0329 (16)	0.064 (2)	0.048 (2)	−0.0001 (17)	0.0059 (14)	−0.0020 (18)
C21	0.045 (2)	0.073 (3)	0.072 (3)	0.0121 (19)	0.0091 (19)	−0.001 (2)
C22	0.0365 (16)	0.059 (2)	0.0399 (19)	−0.0068 (15)	−0.0020 (14)	0.0013 (16)
C23	0.0337 (15)	0.0435 (18)	0.0392 (19)	−0.0033 (14)	0.0029 (13)	−0.0046 (15)
C24	0.0333 (15)	0.060 (2)	0.0395 (19)	−0.0020 (16)	−0.0018 (14)	−0.0030 (17)
C25	0.0447 (19)	0.070 (2)	0.055 (2)	−0.0174 (19)	0.0021 (16)	−0.003 (2)
C26	0.0396 (18)	0.056 (2)	0.051 (2)	−0.0045 (16)	−0.0047 (16)	−0.0079 (18)
C27	0.0354 (15)	0.0500 (19)	0.0389 (19)	−0.0004 (15)	−0.0024 (14)	−0.0075 (16)
C28	0.052 (2)	0.078 (3)	0.049 (2)	−0.0049 (19)	0.0043 (17)	0.002 (2)
C29	0.054 (2)	0.080 (3)	0.052 (2)	0.000 (2)	−0.0118 (18)	0.012 (2)
C30	0.0357 (19)	0.128 (4)	0.058 (3)	−0.018 (2)	0.0005 (18)	0.010 (3)

### Geometric parameters (Å, °)

**Table:**

O1—C1	1.197 (4)	C14—H14A	0.9700
O2—C1	1.328 (4)	C14—H14B	0.9700
O2—H2A	0.91 (6)	C15—C16	1.487 (5)
O3—C13	1.225 (4)	C16—C30	1.312 (5)
O4—C15	1.203 (4)	C16—C17	1.515 (4)
O5—C15	1.318 (4)	C17—C24	1.525 (4)
O5—H5A	0.90 (5)	C17—C18	1.526 (4)
O6—C26	1.233 (4)	C17—H17A	0.9800
C1—C2	1.480 (5)	C18—C19	1.524 (5)
C2—C29	1.314 (5)	C18—H18A	0.9700
C2—C3	1.520 (4)	C18—H18B	0.9700
C3—C4	1.530 (4)	C19—C20	1.523 (5)
C3—C10	1.537 (4)	C19—H19A	0.9700
C3—H3A	0.9800	C19—H19B	0.9700
C4—C5	1.487 (4)	C20—C21	1.508 (5)
C4—H4A	0.9700	C20—C22	1.550 (5)
C4—H4B	0.9700	C20—H20A	0.9800
C5—C11	1.341 (4)	C21—H21A	0.9600
C5—C6	1.512 (4)	C21—H21B	0.9600
C6—C7	1.534 (5)	C21—H21C	0.9600
C6—C14	1.545 (5)	C22—C23	1.509 (4)
C6—H6A	0.9800	C22—C25	1.537 (4)
C7—C8	1.522 (6)	C22—H22A	0.9800
C7—C9	1.530 (5)	C23—C27	1.342 (4)
C7—H7A	0.9800	C23—C24	1.496 (4)
C8—H8A	0.9600	C24—H24A	0.9700
C8—H8B	0.9600	C24—H24B	0.9700
C8—H8C	0.9600	C25—C26	1.492 (5)
C9—C10	1.523 (5)	C25—H25A	0.9700
C9—H9A	0.97 (4)	C25—H25B	0.9700
C9—H9B	0.97 (3)	C26—C27	1.459 (4)
C10—H10A	0.9700	C27—C28	1.492 (5)
C10—H10B	0.9700	C28—H28A	0.9600
C11—C13	1.454 (5)	C28—H28B	0.9600
C11—C12	1.486 (5)	C28—H28C	0.9600
C12—H12A	0.9600	C29—H29A	0.9300
C12—H12B	0.9600	C29—H29B	0.9300
C12—H12C	0.9600	C30—H30B	0.9300
C13—C14	1.489 (5)	C30—H30C	0.9300
			
C1—O2—H2A	109 (3)	O5—C15—C16	114.1 (3)
C15—O5—H5A	115 (3)	C30—C16—C15	119.8 (3)
O1—C1—O2	120.8 (4)	C30—C16—C17	125.6 (3)
O1—C1—C2	124.8 (4)	C15—C16—C17	114.6 (3)
O2—C1—C2	114.3 (3)	C16—C17—C24	111.4 (2)
C29—C2—C1	115.3 (3)	C16—C17—C18	109.3 (3)
C29—C2—C3	125.2 (3)	C24—C17—C18	112.5 (3)
C1—C2—C3	119.5 (3)	C16—C17—H17A	107.8
C2—C3—C4	111.1 (3)	C24—C17—H17A	107.8
C2—C3—C10	112.3 (3)	C18—C17—H17A	107.8
C4—C3—C10	111.1 (3)	C19—C18—C17	115.7 (3)
C2—C3—H3A	107.4	C19—C18—H18A	108.4
C4—C3—H3A	107.4	C17—C18—H18A	108.4
C10—C3—H3A	107.4	C19—C18—H18B	108.4
C5—C4—C3	117.3 (3)	C17—C18—H18B	108.4
C5—C4—H4A	108.0	H18A—C18—H18B	107.4
C3—C4—H4A	108.0	C20—C19—C18	116.7 (3)
C5—C4—H4B	108.0	C20—C19—H19A	108.1
C3—C4—H4B	108.0	C18—C19—H19A	108.1
H4A—C4—H4B	107.2	C20—C19—H19B	108.1
C11—C5—C4	122.2 (3)	C18—C19—H19B	108.1
C11—C5—C6	113.1 (3)	H19A—C19—H19B	107.3
C4—C5—C6	124.8 (3)	C21—C20—C19	112.0 (3)
C5—C6—C7	115.5 (3)	C21—C20—C22	112.9 (3)
C5—C6—C14	102.9 (3)	C19—C20—C22	113.2 (3)
C7—C6—C14	113.0 (3)	C21—C20—H20A	106.0
C5—C6—H6A	108.4	C19—C20—H20A	106.0
C7—C6—H6A	108.4	C22—C20—H20A	106.0
C14—C6—H6A	108.4	C20—C21—H21A	109.5
C8—C7—C9	112.2 (3)	C20—C21—H21B	109.5
C8—C7—C6	112.0 (3)	H21A—C21—H21B	109.5
C9—C7—C6	113.1 (3)	C20—C21—H21C	109.5
C8—C7—H7A	106.3	H21A—C21—H21C	109.5
C9—C7—H7A	106.3	H21B—C21—H21C	109.5
C6—C7—H7A	106.3	C23—C22—C25	102.8 (3)
C7—C8—H8A	109.5	C23—C22—C20	112.0 (3)
C7—C8—H8B	109.5	C25—C22—C20	112.5 (3)
H8A—C8—H8B	109.5	C23—C22—H22A	109.8
C7—C8—H8C	109.5	C25—C22—H22A	109.8
H8A—C8—H8C	109.5	C20—C22—H22A	109.8
H8B—C8—H8C	109.5	C27—C23—C24	122.9 (3)
C10—C9—C7	117.1 (3)	C27—C23—C22	112.7 (3)
C10—C9—H9A	105 (2)	C24—C23—C22	124.4 (3)
C7—C9—H9A	111 (2)	C23—C24—C17	116.9 (3)
C10—C9—H9B	111 (2)	C23—C24—H24A	108.1
C7—C9—H9B	109 (2)	C17—C24—H24A	108.1
H9A—C9—H9B	103 (3)	C23—C24—H24B	108.1
C9—C10—C3	114.0 (3)	C17—C24—H24B	108.1
C9—C10—H10A	108.7	H24A—C24—H24B	107.3
C3—C10—H10A	108.7	C26—C25—C22	105.0 (3)
C9—C10—H10B	108.7	C26—C25—H25A	110.7
C3—C10—H10B	108.7	C22—C25—H25A	110.7
H10A—C10—H10B	107.6	C26—C25—H25B	110.7
C5—C11—C13	109.3 (3)	C22—C25—H25B	110.7
C5—C11—C12	127.4 (3)	H25A—C25—H25B	108.8
C13—C11—C12	123.2 (3)	O6—C26—C27	125.0 (3)
C11—C12—H12A	109.5	O6—C26—C25	126.4 (3)
C11—C12—H12B	109.5	C27—C26—C25	108.6 (3)
H12A—C12—H12B	109.5	C23—C27—C26	109.1 (3)
C11—C12—H12C	109.5	C23—C27—C28	127.4 (3)
H12A—C12—H12C	109.5	C26—C27—C28	123.5 (3)
H12B—C12—H12C	109.5	C27—C28—H28A	109.5
O3—C13—C11	125.1 (3)	C27—C28—H28B	109.5
O3—C13—C14	125.7 (4)	H28A—C28—H28B	109.5
C11—C13—C14	109.2 (3)	C27—C28—H28C	109.5
C13—C14—C6	105.4 (3)	H28A—C28—H28C	109.5
C13—C14—H14A	110.7	H28B—C28—H28C	109.5
C6—C14—H14A	110.7	C2—C29—H29A	120.0
C13—C14—H14B	110.7	C2—C29—H29B	120.0
C6—C14—H14B	110.7	H29A—C29—H29B	120.0
H14A—C14—H14B	108.8	C16—C30—H30B	120.0
O4—C15—O5	122.7 (3)	C16—C30—H30C	120.0
O4—C15—C16	123.1 (3)	H30B—C30—H30C	120.0
			
O1—C1—C2—C29	−0.4 (6)	O4—C15—C16—C30	−159.5 (4)
O2—C1—C2—C29	178.2 (3)	O5—C15—C16—C30	21.6 (5)
O1—C1—C2—C3	178.2 (4)	O4—C15—C16—C17	20.0 (5)
O2—C1—C2—C3	−3.1 (4)	O5—C15—C16—C17	−159.0 (3)
C29—C2—C3—C4	13.2 (5)	C30—C16—C17—C24	11.7 (5)
C1—C2—C3—C4	−165.3 (3)	C15—C16—C17—C24	−167.7 (3)
C29—C2—C3—C10	−111.9 (4)	C30—C16—C17—C18	−113.2 (4)
C1—C2—C3—C10	69.6 (4)	C15—C16—C17—C18	67.4 (4)
C2—C3—C4—C5	166.6 (3)	C16—C17—C18—C19	−152.4 (3)
C10—C3—C4—C5	−67.6 (4)	C24—C17—C18—C19	83.4 (4)
C3—C4—C5—C11	−173.6 (3)	C17—C18—C19—C20	−66.8 (5)
C3—C4—C5—C6	6.0 (5)	C18—C19—C20—C21	−67.0 (4)
C11—C5—C6—C7	−121.7 (3)	C18—C19—C20—C22	62.1 (4)
C4—C5—C6—C7	58.7 (5)	C21—C20—C22—C23	51.2 (4)
C11—C5—C6—C14	2.0 (4)	C19—C20—C22—C23	−77.3 (4)
C4—C5—C6—C14	−177.7 (3)	C21—C20—C22—C25	−63.9 (4)
C5—C6—C7—C8	53.1 (4)	C19—C20—C22—C25	167.5 (3)
C14—C6—C7—C8	−65.1 (4)	C25—C22—C23—C27	11.9 (4)
C5—C6—C7—C9	−74.9 (4)	C20—C22—C23—C27	−109.1 (3)
C14—C6—C7—C9	167.0 (3)	C25—C22—C23—C24	−169.8 (3)
C8—C7—C9—C10	−64.2 (5)	C20—C22—C23—C24	69.2 (4)
C6—C7—C9—C10	63.7 (5)	C27—C23—C24—C17	171.4 (3)
C7—C9—C10—C3	−68.2 (5)	C22—C23—C24—C17	−6.7 (5)
C2—C3—C10—C9	−150.9 (3)	C16—C17—C24—C23	176.3 (3)
C4—C3—C10—C9	84.1 (4)	C18—C17—C24—C23	−60.6 (4)
C4—C5—C11—C13	179.8 (3)	C23—C22—C25—C26	−13.2 (4)
C6—C5—C11—C13	0.2 (4)	C20—C22—C25—C26	107.4 (3)
C4—C5—C11—C12	0.6 (6)	C22—C25—C26—O6	−169.9 (4)
C6—C5—C11—C12	−179.0 (4)	C22—C25—C26—C27	11.0 (4)
C5—C11—C13—O3	177.8 (4)	C24—C23—C27—C26	176.4 (3)
C12—C11—C13—O3	−3.0 (6)	C22—C23—C27—C26	−5.3 (4)
C5—C11—C13—C14	−2.4 (4)	C24—C23—C27—C28	−5.9 (5)
C12—C11—C13—C14	176.9 (4)	C22—C23—C27—C28	172.4 (3)
O3—C13—C14—C6	−176.7 (4)	O6—C26—C27—C23	177.0 (3)
C11—C13—C14—C6	3.5 (4)	C25—C26—C27—C23	−3.9 (4)
C5—C6—C14—C13	−3.2 (4)	O6—C26—C27—C28	−0.8 (5)
C7—C6—C14—C13	122.1 (3)	C25—C26—C27—C28	178.3 (3)

### Hydrogen-bond geometry (Å, °)

**Table:**

*D*—H···*A*	*D*—H	H···*A*	*D*···*A*	*D*—H···*A*
O2—H2A···O6	0.91 (6)	1.80 (6)	2.699 (4)	166 (5)
O5—H5A···O3^i^	0.90 (5)	1.76 (5)	2.658 (4)	171 (5)

Symmetry codes: (i) *x*+2, *y*, *z*+1.
